# Interventions aimed at improving the nursing work environment: a systematic review

**DOI:** 10.1186/1748-5908-5-34

**Published:** 2010-04-27

**Authors:** Donna MJ Schalk, Marloes LP Bijl, Ruud JG Halfens, Louk Hollands, Greta G Cummings

**Affiliations:** 1 Faculty of Health and Social Studies, Research Department Acute Care, Han University of Applied Sciences, Nijmegen, the Netherlands; 2 Department of Healthcare and Nursing Science, Maastricht University, Maastricht, the Netherlands; 3 Faculty of Nursing, University of Alberta, Edmonton, Alberta, Canada

## Abstract

**Background:**

Nursing work environments (NWEs) in Canada and other Western countries have increasingly received attention following years of restructuring and reported high workloads, high absenteeism, and shortages of nursing staff. Despite numerous efforts to improve NWEs, little is known about the effectiveness of interventions to improve NWEs. The aim of this study was to review systematically the scientific literature on implemented interventions aimed at improving the NWE and their effectiveness.

**Methods:**

An online search of the databases CINAHL, Medline, Scopus, ABI, Academic Search Complete, HEALTHstar, ERIC, Psychinfo, and Embase, and a manual search of Emerald and Longwoods was conducted. (Quasi-) experimental studies with pre/post measures of interventions aimed at improving the NWE, study populations of nurses, and quantitative outcome measures of the nursing work environment were required for inclusion. Each study was assessed for methodological strength using a quality assessment and validity tool for intervention studies. A taxonomy of NWE characteristics was developed that would allow us to identify on which part of the NWE an intervention targeted for improvement, after which the effects of the interventions were examined.

**Results:**

Over 9,000 titles and abstracts were screened. Eleven controlled intervention studies met the inclusion criteria, of which eight used a quasi-experimental design and three an experimental design. In total, nine different interventions were reported in the included studies. The most effective interventions at improving the NWE were: primary nursing (two studies), the educational toolbox (one study), the individualized care and clinical supervision (one study), and the violence prevention intervention (one study).

**Conclusions:**

Little is known about the effectiveness of interventions aimed at improving the NWE, and published studies on this topic show weaknesses in their design. To advance the field, we recommend that investigators use controlled studies with pre/post measures to evaluate interventions that are aimed at improving the NWE. Thereby, more evidence-based knowledge about the implementation of interventions will become available for healthcare leaders to use in rebuilding nursing work environments.

## Background

The work environment of nurses in Canada has increasingly received attention due to high absenteeism and shortages of nursing staff, augmented by dramatic cutbacks and restructuring of healthcare services in the 1990s. The restructuring led to forced layoff of large numbers of nurses in short time periods [1], higher nurse/patient ratios, reduced professional and clinical support, and an increase in non-nursing tasks for nurses [2]. These developments led to deteriorated work environments for nurses, and many nurses are retiring early or leaving the profession because of stressful working conditions [3]. The challenges faced by Canadian nurses are not unique to Canada; most of the Western world countries face similar problems [2]. Because the nursing workforce is one of the most important factors in the healthcare system in providing safe patient care [4], it is crucial to improve their work environments, especially to keep up with the increasing patient numbers and demands due to the aging population.

Much has been written about interventions to improve the nursing work environment (NWE). However, most of these studies provide advice on work environment interventions and do not report actual implementation or effectiveness of interventions. Moreover, we could not find any reviews that evaluated the effects of implementing nursing work environment interventions. Therefore, the aim of this study was to review systematically the scientific literature on implemented interventions aimed at improving the NWE and their effectiveness. This study was guided by two research questions: Which interventions have been implemented to improve the nursing work environment? How effective are these interventions at improving the nursing work environment?

### Theoretical background

To systematically review the effects of interventions on NWEs, we first needed to identify what constitutes a NWE, as it is comprised of multiple characteristics and therefore cannot be measured by one single outcome measure. For that reason--preliminary to the systematic review--a literature review was conducted to explore the concept of NWE, identify available conceptual frameworks, and construct a taxonomy of NWE characteristics. In order to answer the research questions, this taxonomy allowed us to identify which characteristic of the NWE an intervention was focused on so we could then examine the effects of the intervention.

Several NWE conceptual frameworks were found [5-9]. However, further preliminary exploration of the literature showed that the NWE consists of more characteristics than described by the frameworks. Therefore, the frameworks were not sufficiently comprehensive to use, and more literature was screened to create a taxonomy of reported NWE characteristics.

In examining the literature on NWE, some inclusion and exclusion criteria were used. The focus was only on the environment in which nurses work and not on personal characteristics of nurses such as their experience, stress levels, work-life balance, self-image, and life values. Furthermore, we distinguished work environment characteristics from work environment indicators, such as job satisfaction, decreased turnover, absenteeism, or burn-out. The literature search was based on the most recent papers (2008) on NWEs and their references back to the first paper published in this field (1987). A content analysis was performed where we sorted and clustered the NWE characteristics into a taxonomy. We continued to search the literature until the retrieved NWE characteristics were saturated and no new characteristics were obtained. Table 1 shows the taxonomy of NWE characteristics referred to in the literature. The characteristics of a NWE that were defined in this study are: teamwork, leadership, autonomy, workload, clarity, recognition, physical comfort, flexible scheduling, organizational policies, professional development opportunities, salary, participation in decision making, innovation, and workplace safety.

**Table 1:** Taxonomy of NWE characteristics.

Nursing work environment	
**Characteristics**	**Synonyms**

Teamwork [14,17]	Positive work relationships [8,19,10]
	Interprofessional relations [6,9,10]
	Peer cohesion [5]
	Social support [13]
	Collaborative decision making [16]
	Clinical support [15]
	Communication [10,14,17]

Leadership [6,7,9,13-18]	Supervisor support [5,9,12,14,19]
	Communication [6,8,10,14,17]
	Feedback [10,14]

Autonomy [5,6,8,10,14,18,19]	Empowering [16]
	Professional identity [12]

Workload [6,8,11,13-15,18]	Adequate staffing [6,10,11,14,17,19]
	Work pressure [5,12]

Clarity [5,8,14]	Degree of role specificity [6]

Recognition [6,7,10,12,16]	Respect [6,10,15,17]
	Reward systems [7,9,12]

Physical comfort [5]	Availability of equipment, materials, supplies and other non-human resources [6,9,10,14]
	Work design [7]

Flexible scheduling [6,8,10-12,14,15]	

Organizational policies [6]	Characteristics of the organization [10]
	Organizational stability [8]
	Organizational culture [7,18]

Professional development opportunities [8,16,18]	Opportunities for personal growth [7,10]
	Career development [12]
	Career laddering [6,15]
	Educational opportunities [11-13,15-17,19]

Salary [6,10,13]	Salary benefits [6,10]

Participation in decision making [7,10,14,16,18]	

Innovation [5]	Technological demands [6,11]

Workplace safety [8,12-14,17]	Absence of violence [12] Protection against violence [15]

The 14 reported NWE characteristics were all consistently found in the literature, sometimes with different synonyms (Table 1). Teamwork was mostly defined as positive work relationships among the nurses or other personnel, but also as interprofessional relations, peer cohesion, social support, collaborative decision-making, and the amount of clinical support. Leadership was reported in all except one study and often was explained as the amount of support or feedback received from the supervisor, the communication with the leader, and leadership style. Autonomy was very consistently used in the literature and referred to how autonomous or empowered nurses felt in their work. Workload was also frequently reported in the literature as a NWE issue, often directly related to staffing; where adequate staffing is perceived to reduce the work pressure and workload of nurses. Clarity refers to the extent to which employees know what to expect in their daily routine (role clarity) and how explicitly rules and policies are communicated (goal clarity). Recognition for their work is highly valued by nurses in terms of respect and rewards received for their job. Also the physical work environment, such as availability of resources and the design of the workplace, adds to the quality of the work environment. Flexible scheduling was reported as an important characteristic contributing to the quality of the work environment because the nurses are more satisfied with working hours when they have a certain influence on them. Several organizational characteristics, including culture and stability of the organization, were identified as contributing to the NWE as they shape the environment in which nurses work. One of the major NWE work environment characteristics was the opportunity for professional development, which includes personal growth, career development/laddering, and education. Furthermore, nurses indicated that a good wage (salary) was an important characteristic in their work environment. Participation in decision making was found important, defined as nurses having the voice and ability to participate in organizational or clinical decision-making. Innovation referred to the degree of variety, change, and new approaches of which technological advances was one form. Workplace safety has been a major issue in the NWE in recent years where violence by patients against nurses is reportedly more prevalent. The absence or prevention of violence in the work environment of nurses contributes to a safer and higher quality NWE.

The NWE characteristics reported in the taxonomy in Table 1 were addressed by studies in different ways. The NWE characteristics were identified to: develop a work environment scale [5]; prioritize nursing worklife issues defined by nurses for nurses [10-13]; develop a unifying framework of nursing worklife issues or healthy work environments [6,8]; address worklife concerns or issues of nurses [14,15]; be hallmarks or critical factors for a professional nursing practice environment and achieving work environment excellence [7,16-18]; be essential attributes for quality care [19]; or create a program for staff nurses to improve the workforce environment [9].

To conclude, the NWE consists of these characteristics that we deemed in this systematic review to be the dependent variables. The independent variables examined in this systematic review are the interventions aimed at improving the NWE.

## Methods

### Search methods

A list of initial search terms was agreed upon by the authors (DMJS, MLPB, and GGC). Then, a preliminary scoping literature review followed to give information about interventions that were implemented in the scope of NWEs, and to identify relevant search terms to add to the initial search term list. In this way, we were assured that the search terms would cover all possible relevant studies. The final search terms (practice environment, work environment, worklife, work life, workplace, working conditions, work climate, innovation, intervention, organizational improvement, strategies, strategy, and nurs*) were used for the online search of the following electronic bibliographic databases: CINAHL, Medline, Scopus, ABI, Academic Search Complete, HEALTHstar, ERIC, Psychinfo, and Embase. The same search terms were used in the manual search of Longwoods (online publisher of healthcare papers), Emerald (online publisher of business and management research), and a dissertation database of doctoral and masters theses from 1,000 North American graduate schools and European universities. The detailed search strategy is presented in Additional file 1.

### Inclusion and exclusion criteria

Studies published in English between 1985 and April 2008 that met the following inclusion criteria were reviewed: the study population consisted of nurses (licensed practical nurses, registered nurses, nursing attendants/aides/assistants, and student nurses); an intervention was implemented to improve the work environment of the nurses; pre/post implementation measures were performed; the study used both control and intervention groups, and the study reported quantitative outcome measures of the work environment of nurses. All studies, except doctoral dissertations, had to be published in the peer-reviewed literature.

### Screening

After removal of duplicates, the first two authors (DMJS and MLPB) each screened one-half of the titles of the studies using the inclusion criteria. Studies that were clearly not relevant based on the title alone were excluded. Doubtful titles were discussed between the two authors until consensus was reached. Next, the abstracts of the remaining studies were divided between the two authors, and each screened for presence of an intervention to improve the work environment of nurses. When it was not completely clear if the study contained an intervention to improve the NWE the author included the study. Then, the studies were fully read and screened by both authors for an intervention to improve the work environment of nurses and pre/post measure. Together with the senior author (GGC), a further selection of studies was made, which was screened on all inclusion criteria. There was no disagreement regarding eligibility between the authors in selecting studies for the review.

### Quality assessment

The studies that met the inclusion criteria were assessed for methodological strength using the quality assessment and validity tool for intervention studies, originated from Estabrooks *et al*. [20] and adapted by Cummings *et al*. [21]. The instrument used 13 items to evaluate the sampling, design, measurement, statistical analysis, and drop outs. Each item was scored as zero or one, except for two items: 'use of matching' and 'use of several post-test measures' were scored with zero, one, or two points, resulting in a maximum possible score of 15 points. The total number of points that the study scored was divided by 15. Studies that scored <0.50 were rated as weak, 0.50 to 0.74 were rated as moderate, and studies that scored >0.75 were rated as strong. The weak studies were excluded to reduce bias in the integration of study results, and the moderate and strong studies were included for the final data extraction. Each study was independently rated by two reviewers. When changes in the assessment were found, the researchers discussed the discrepancy together with a third reviewer until consensus was reached.

### Data extraction

The following data were extracted from the studies in the final inclusion group by two reviewers: author, year, country, design type, intervention format/setting, intervention duration, intervention provider, recipients of intervention, fidelity of intervention implementation, content/elements, NWE targeted, and quality score.

### Data synthesis

Data from the included studies were synthesized by determining whether a significant change in the NWE outcome resulted from the implemented intervention. Only the outcome measures in the included studies that could be categorized to the NWE characteristics in the taxonomy were analyzed. Furthermore, we examined if the reported differences in the outcome measures were relative to the control group or to the pre-intervention measure. If the study reported outcomes that were both relative to the control group as well as to the pre-intervention measure, only the outcomes relative to the control group were reported. When a study used several intervention/control groups, significant improvements were reported if at least one of the intervention groups showed a significant result compared to the control groups.

## Results

### Search results

**Figure 1:**
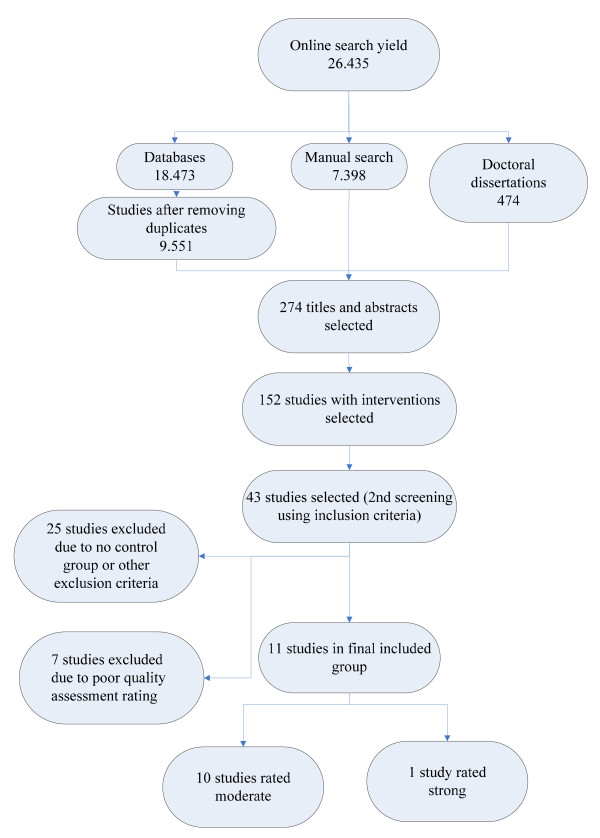
**Search and retrieval process**.

With the final search terms, a total of 26,435 titles and abstracts were retrieved and screened solely for the presence on an intervention to improve the work environment of nurses. This identified 274 studies that were retrieved and screened for interventions to improve the work environment of nurses and for pre/post measure designs. After this, 152 studies remained that were fully read and screened using the inclusion criteria. This led to 43 studies selected that were assessed on their quality. After exclusion of studies with weak designs, 11 controlled intervention studies, of which one was rated as strong and ten were rated as moderate, remained for data extraction. An overview of the search and retrieval process can be found in Figure 1. A list of the excluded studies and reasons for exclusion is presented in Additional file 2.

Data of the final 11 included studies and doctoral dissertations in this systematic review are presented in Additional file 2 and consisted of studies from the United States (5), The Netherlands (2), Sweden (3) and Norway (1). All studies were either quantitative or had a mixed method design, and were published between 1989 and 2007. All studies reported the demographics of the study objects; however, one study gave no information about the mean age of participants. Mean age in the 10 remaining intervention studies ranged from 31.7 to 42.5.

A total of 1,833 participants were included in all studies of this review. The study subjects were mostly referred to as registered nurses or licensed practical nurses. Others were described as nursing technicians, nursing attendants/aides/assistants, student nurse extenders, secretaries, and unit leaders. All except one study reported the participants' gender, with percentages of females ranging from 72% to 100%.

Five studies were conducted in hospital settings, three studies were conducted in nursing homes, one study described the implementation of an intervention in a community healthcare institution, one study took place in a psychogeriatric clinic, and one reported a study in several healthcare work-places, representing emergency departments, geriatric, psychiatric, and home healthcare sites. Duration of the interventions ranged from one month to three years. The interventions were delivered by external providers (researchers or psychologists) or internal facilitators (nurse managers or supervisors). We also examined the studies on intervention fidelity, which refers to whether the intervention was delivered as intended [22]. However, only two studies reported some information related to difficulties in the implementation process due to organizational problems. The characteristics of included studies are presented in Additional file 3.

### Quality assessment

A summary of the quality assessment of included studies is presented in Additional file 4. Of the 11 included controlled studies, eight used a quasi-experimental design and three used an experimental design. Furthermore, six of the 11 studies used a pretest/posttest design with repeated measures; the remaining studies measured the outcomes only once before and after the intervention. Only two of the 11 studies included in this review used probability sampling. Eight studies did not have an appropriate or justified sample size. Missing data were managed appropriately through statistical analyses in one study; one study reported that there were no missing data, and nine studies did not report missing data or statistical analyses to adjust for the missing data. Furthermore, six of the 11 studies did not report information about the validity of the instruments to measure the NWE.

Ten studies reported some form of reliability or internal consistency. In all studies, the statistical analysis was appropriate for the main study outcome and more than 80% of other results. Also, p-values and confidence intervals in all 11 studies were properly reported. Only three studies stated clearly that the groups were matched on sample characteristics such as gender, activity, or age by means of randomization to control confounders. The remaining studies still scored one point on this item because they used both a control and intervention group.

### Implemented interventions and their effectiveness

The research questions of this systematic review aimed to identify which interventions have been implemented to improve the NWE and how effective they were at improving the NWE. Table 2 presents the interventions of the studies included in this systematic review and the reported outcome measures. In this table, the outcome measures used by the studies are linked to the NWE characteristics of the taxonomy presented in the theoretical background. Additional data of the implementation of the interventions are presented in Additional file 3.

**Table 2:** Implemented interventions and their effectiveness.

Intervention	NWE taxonomy characteristics	Outcome measure used	Significance of the outcome measures (p ≤ 0.05)	Differences relative to control group or pre-intervention
Primary nursing [23,24]	Teamwork	Social support [23]	+	I-C
		Communication [23]	-	I-C
				
	Leadership	Leadership style [24]	NS	I-C
	Autonomy	Autonomy [24]	+	I-C
		Job autonomy [23]	+	I-C
	Workload	Complexity [24]	NS	I-C
		Job demands [23]	+	I-C
	Clarity	Feedback/clarity [24]	NS	I-C
		Resident assignment [23]	+	I-C

Shared governance [25]	Teamwork	Co-worker support [25]	NS	I-C
		Intrapersonal conflict [25]	-	I-C
	Autonomy	Autonomy [25]	NS	I-C
	Clarity	Role ambiguity [25]	NS	I-C
		Role conflict [25]	NS	I-C

Social support training and stress inoculation training [26]	Teamwork	Peer cohesion [26]	+	I-C
	Leadership	Supervisor support [26]	+	I-C

Short-term participatory intervention [27]	Teamwork	Social support [27]	+	I-C
		Team style [27]	NS	I-C
				
	Leadership	Management relations and style [27]	NS	I-C
		Consideration for individuals [27]	NS	I-C
	Autonomy	Decision authority [27]	NS	I-C
		Autonomy & responsibility [27]	NS	I-C
	Workload	Job demands [27]	+	I-C
	Clarity	Guidelines on how to do the job [27]	+	I-C
	Professional development opportunities	Time to develop [27]	NS	I-C
		Opportunity to develop [27]	+	I-C

Nursing practice quality circle [28]	Teamwork	Peer cohesion [28]	NS	I-C
	Leadership	Supervisor support [28]	NS	I-C
		Control [28]	NS	I-C
	Autonomy	Autonomy [28]	NS	I-C
	Workload	Work pressure [28]	+	I-C
	Clarity	Clarity [28]	NS	I-C
	Physical comfort	Physical comfort [28]	NS	I-C
	Innovation	Innovation [28]	+	I-C

Educational toolbox [29]	Teamwork	Work-climate [29]	NS	I-C
	Leadership	Leadership [29]	+	I-C
		Performance feedback [29]	+	I-C
	Autonomy	Participation [29]	+	I-C
	Clarity	Goal clarity [29]	NS	I-C
	Professional development opportunities	Skills development [29]	+	I-C
	Participation in decision making	Participation [29]	+	I-C

Individualized care and regular systematic clinical supervision [30]	Teamwork	Cooperation [30]	+	P-P
	Autonomy	Autonomy [30]	+	P-P
	Recognition	Recognition [30]	+	P-P
	Professional development opportunities	Professional growth [30]	+	P-P

Supervisor positive feedback training [31]	Teamwork	Peer cohesion [31]	NS	I-C
	Leadership	Supervisor support [31]	NS	I-C

Violence prevention intervention [32,33]	Workplace safety	Registration violent events [32]	-	I-C
		Awareness of risk situations for violence [32]	+	I-C
		Avoidance of potential dangerous situations [32]	+	I-C
		Dealing with aggressive patients [32]	+	I-C
		Perceived knowledge [33]	+	I-C
		Self-efficacy [33]	+	I-C
		Violence prevention skills [33]	+	I-C

+ intervention led to significant improvement in the outcome measure

- intervention led to significant deterioration of the outcome measure

NS No significant effect was found

I-C Results of the intervention group are compared with the results of the control group

P-P Results of the intervention group on the post measure are compared with the results of the intervention group on the pre measure.

In total, nine different interventions were reported in the included studies. Two studies [23,24] reported the intervention 'primary nursing,' which consisted of the assignment of patients to primary nurses. These primary nurses were responsible for the total nursing care of their patients and received special support on how to deal with the higher demands for autonomy in their work. Primary nursing showed mixed effects on improving the NWE. Significant improvements were made in autonomy [23,24], workload [23], clarity [23], and teamwork [23,24]. The only significant negative effect was found in the communication among nurses [23].

One study [25] reported the implementation of shared governance; nurses from patient units in an acute care setting were offered an organizational framework that offered them maximal participation in decisions about work and the workplace. Shared governance had no significant effects on nurses' autonomy, teamwork, and clarity of work, and even showed a significant deterioration of intrapersonal relationships through increased conflict (perceived difficulties in interactions between members of the same unit or department) [25].

Toloczko [26] examined the implementation of stress inoculation training and social support training given by psychologists. The training proposed the acquisition of sufficient knowledge, self-understanding, and coping skills to facilitate the nurses working in the hospital with better ways of handling stressful events. Social support training and stress inoculation training significantly improved the leadership and nurses' teamwork [26].

In a short-term participatory intervention [27], employees of two healthcare institutions collectively created a plan that would improve their work environment, which they implemented with their own work group by focusing on certain elements of the workplace that needed improvement. The intervention was successful in significantly improving clarity, (decision authority), workload, teamwork, and professional development opportunities [27].

One study [28] reported the implementation of nursing practice quality circles (NPQC)--groups of nurses from one unit met once a week on work-time to identify and select problems, analyze causes, recommend solutions to management, and when possible, implement solutions. Furthermore, the NPQC received training in specific techniques of brainstorming, data collection, decision analysis, sampling, cause-and-effect analysis, and group task and group maintenance functions. The study showed a significant improvement in the NWE taxonomy characteristics workload and innovation in the workplace (both decreased) [28].

In the study of Arnetz and Hasson [29], a workgroup of researchers and management representatives collated an educational toolbox of practical instruments for use at elderly care workplaces. The toolbox instruments were meant to improve nursing staff knowledge in specific areas or designed to help nursing staff in various aspects of their daily work. The educational toolbox improved nurses' autonomy, teamwork, leadership, professional development opportunities, and participation in decision making [29].

An intervention to improve the work environment of nurses working in a psychogeriatric clinic consisted of the implementation of individualized care and regular systematic clinical supervision [30]. Rigor in planning of the care was believed to support the nurses' interpretation of what was best for the patient. Regular systematic clinical supervision was implemented to support the nurses and relieve them of their emotional strain stemming from their work [30]. This intervention showed significant improvements in nurses' autonomy, teamwork, professional development opportunities, and recognition for their work [30].

Supervisor positive feedback training [31] was the intervention in another study, in which supervisors of nurses received advice from researchers on how to give more positive feedback and were encouraged to adjust their supervision styles. The intervention showed no significant effects on teamwork and leadership [31].

Two studies described a violence prevention intervention [32,33]. In one of these studies, workplace routines were established in various healthcare settings for managing and reducing violent incidents towards healthcare staff [32]. The other study consisted of training, based on social cognitive theory, in which nurses from nursing homes were taught to use violence prevention skills [33]. The intervention improved workplace safety by increasing awareness, prevention, and skills of violence management [32,33]. However, the reported violence significantly increased in the intervention group [32].

In general, when looking at the combination of the number of outcome measures per intervention and the amount of significant improvements per intervention, primary nursing (56%), the educational toolbox (71%), the individualized care and clinical supervision (100%), and the violence prevention intervention (86%) were most effective in improving the NWE. The remaining interventions showed effectiveness of ≤ 50%.

## Discussion

### Quality of studies

It is clear that a body of literature exists about interventions to improve the NWE, but many studies were excluded from this systematic review due to weaker research designs, lack of control groups, pre/post measures, or sufficient sample size. This resulted in only 11 studies with (quasi-) experimental designs that could be included for analysis. Study weaknesses threaten the quality of the evidence and bias assessment of effectiveness of the interventions. Despite excluding all studies assessed as weak, six studies still remained that did not report the validity or reported insufficient information about the validity of the instruments to measure the NWE. This increases the risk of instruments not measuring the specific NWE characteristic they purport to, which could lead to biased results [34]. Eight studies used a quasi-experimental design, and only three studies used randomization [26,30,32]. The absence of randomization suggests the use of nonequivalent control, leading to greater risk of confounding factors influencing the reported effect on the NWE. However, we argue that the use of randomization in studies in healthcare settings is not frequently used due to practical limitations that are inevitably linked to these settings. It can be difficult to test an intervention randomly on one group of nurses, and not on others [34], due to collective agreements, policies, costs, or ethics. Cluster randomization of nursing units to intervention and control conditions is possible; however, much more costly due to increased study scope required to achieve power to detect an effect. With current nursing shortages and staffing challenges, hospitals may have chosen nursing units that were able and willing to participate. Therefore, quasi-experiments with pre/post measures present a more practical research design for studying the effects of intervention on the work environment of nurses.

Furthermore, only two studies used probability sampling, suggesting that in most cases persons in the population did not have an equal, independent chance of being selected [34]. Eight studies did not report an appropriate or justified sample size, which reduces generalizability of the results to other populations. None of the studies reported the use of an intra-class correlation to assess appropriateness of aggregating data to the unit or facility level. Only two articles reported some information on the fidelity of implementation of the study intervention. The current staffing challenges facing the nursing profession may also have constrained the incorporation of fully randomized groups, use of probability sampling, or achieving appropriate sample size.

### Implemented interventions and their effectiveness

When looking at the effectiveness of the interventions, most interventions showed mixed effects and reported significant improvements in some of the outcome measures. Only shared governance showed no improvements and even led to significant greater intrapersonal conflicts in the shared governance group compared to the control group. However, Kennerly [25], argued that a heightened awareness of differing values and needs of individual group members was reasonable to expect when faced with new experiences [25]. The mixed effects of the studies make it difficult to say which implemented intervention showed the most improvement in the NWE. It is possible that giving some attention to the NWE is more important than the specific type of intervention (Hawthorne effect). This attention and acknowledgment in itself appeared to lead to improvements in multiple work environment characteristics, such as feelings of being valued, having a voice in decision making and increased awareness of working relationships.

Furthermore, it is notable that three NWE taxonomy characteristics--flexible scheduling, organizational policies, and salary--have not been addressed by controlled studies. Flexible scheduling may be difficult to achieve because the nursing shortage leaves healthcare organizations with little scope to be flexible in working hours. In addition, the issues associated with the nursing workforce are very complex and dynamic, and involve multiple stakeholders, including governments, employers, professional associations, unions, and educators [15]. Therefore, NWE taxonomy characteristics, such as salary and organizational policies, cannot be changed easily because they involve national standards and policies and necessitate multiple layers of negotiation to change. On the contrary, teamwork, leadership, autonomy, and clarity were the NWE taxonomy characteristics most frequently addressed, presumably because of their potential modifiability. Improvements in these NWE characteristics may be more easily achieved by relatively small interventions. An increase in autonomy, for example, can be achieved by changing routines/responsibilities; teamwork can be improved by organizing team meetings. In that way, stakeholders may be more likely to address these NWE taxonomy characteristics instead of focusing on complex issues such as salary and organizational policies. However, it should be noted that the rigorous inclusion criteria used in this systematic review could have excluded studies without controlled design that examined NWE taxonomy characteristics, such as organizational policies and flexible scheduling.

### Strengths and limitations

The strength of this systematic review is in the rigorous review, selection of studies, and quality assessment that led to 11 controlled studies of sufficient quality. Another strength of this review is its focus specifically on the work environment of nurses, instead of on healthcare work settings in general. The limitations relate to assumptions that had to be made, because the NWE as dependent variable is such a broad concept and consists of many characteristics. First, assumptions were made as to whether an outcome measure was part of the NWE or not when categorizing the outcome measures into the taxonomy. Second, the outcome measure used in the studies had to fit to one of the NWE taxonomy characteristics and, based on some overlap, a decision was made about which characteristic was the best fit. Furthermore, because of the rigorous quality assessments that were used, some relevant studies were excluded from the data analysis, which may have contributed to knowledge about interventions to improve the NWE. Therefore, this systematic review may underreport the published number and type of interventions implemented to improve the NWE. Another minor limitation is language bias; only studies published in English were included in the systematic review.

## Summary

Many Western countries are experiencing a crisis in nursing due to the high nursing shortages and subsequent deterioration of work. Although a rich body of literature exists reporting the importance of improving the work environment of nurses, this review shows that evidence to support or refute specific NWE interventions is inconclusive. Therefore, future research in this field is urgently required in which the optimum research design would be controlled studies with pre/post measures. In this way, healthcare leaders can rely on more evidence based research in rebuilding NWEs.

## Competing interests

The authors declare that they have no competing interests.

## Authors' contributions

DMJS and MLPB both conducted this study as part of their Master Thesis. GGC, RJGH, and LH supervised them in their thesis. GGC validated the search design, study selection, data extraction, and quality assessment. GGC, RJGH, and LH validated the data analysis. DMJS and MLPB designed and performed the search, selected the studies, assessed the quality of the studies, extracted the data, analyzed the results, and wrote the manuscript. GGC, RJGH, and LH reviewed the systematic review and participated throughout the writing of the review. All authors read and approved the final manuscript.

## Supplementary Material

Additional file 1 **Search Strategy**. Search terms used in EMBASE, ERIC, HealthSTAR, Psycinfo, ASC, CINAHL, Medline, Scopus and ABI, manual search Longwoods and Emerald and doctoral dissertations. Click here for file

Additional file 2 **Details of excluded studies**. Author, title and reasons for excluding screened studies. Click here for file

Additional file 3 **Characteristics of included studies**. Detailed characteristics of included studies. Click here for file

Additional file 4 **Summary of quality assessments**. individual and summary quality assessment scores for each included study. Click here for file
