# Supplementary material for: Interventions aimed at improving the nursing work environment: a systematic review
Source: Implement Sci. 2010 Apr 27;5:34. doi: 10.1186/1748-5908-5-34 (PMC2876995; doi:10.1186/1748-5908-5-34)
Supplement: Additional file 1 — Search Strategy. Search terms used in EMBASE, ERIC, HealthSTAR, Psycinfo, ASC, CINAHL, Medline, Scopus and ABI, manual search Longwoods and Emerald and doctoral dissertations. [file 1748-5908-5-34-S1.DOC]

Additional file 1

Search Strategy

Search terms used in EMBASE, ERIC, HealthSTAR, Psycinfo, ASC, CINAHL, Medline, Scopus and ABI, manual search Longwoods and Emerald and doctoral dissertations.

1. “practice environment”
2. “work environment”
3. “worklife”
4. “work life”
5. “workplace”
6. “working conditions”
7. “work climate”
8. Innovation
9. Intervention
10. Organizational improvement
11. Strategies
12. Strategy
13. Nurs*
