# Supplementary material for: Interventions aimed at improving the nursing work environment: a systematic review
Source: Implement Sci. 2010 Apr 27;5:34. doi: 10.1186/1748-5908-5-34 (PMC2876995; doi:10.1186/1748-5908-5-34)
Supplement: Additional file 2 — Details of excluded studies. Author, title and reasons for excluding screened studies. [file 1748-5908-5-34-S2.DOC]

Additional file 2

Details of excluded studies

| **First Author**  **& year** | **Title** | **Main reason(s) for exclusion from review** |
| --- | --- | --- |
| Lipscomb  2006 | Violence prevention in the mental health setting: the New York State experience | 1. No pre/post measure |
| McGillis Hall  2008 | Outcomes of interventions to improve hospital nursing work environments | 1. No control group |
| Tommasini  1992 | The impact of a staff support group on the work environment of a specialty unit | 1. Sample too small (N=8) |
| Parsons  2004 | Capacity building for magnetism at multiple levels. A healthy workplace intervention, Part II – An emergency department’s healthy workplace process and outcomes | 1. No control group  2. Sample too small, of which also non-nurse participants |
| Berg  1999 | Effects of systematic clinical supervision on psychiatric nurses’ sense of coherence, creativity, work-related strain, job satisfaction and view of the effects from clinical supervision; a pre-post test design | 1. No control group  2. Sample too small (N=22) |
| Pryce  2006 | Evaluation of an open-rota system in a Danish psychiatric hospital: a mechanism for improving job satisfaction and work-life balance | 1. Low quality assessment score  2. No clear distinction in study participants; unknown if effects were applicable to nurses |
| Lavoie-Tremblay  2005 | Improving the psychosocial work environment | 1. No control group |
| McPhail  2005 | Primary nursing. A randomized crossover trial | 1. No control group  2. Sample too small (N=11) |
| Prater  1993 | The impact of shared governance on nurses’ perceptions of organizational climate (Doctoral dissertation) | 1. No control group |
| Bland Jones  1993 | Shared governance and the nursing practice environment | 1. Low quality assessment score |
| Alcock  1993 | Formative evaluation: implementation of primary nursing | 1. Low quality assessment score |
| Bégat  1997 | Implementation of clinical supervision in a medical department: nurses’ views of the effects | 1. Low quality assessment score |
| Petterson  1998 | Psychosocial stressors and well-being in health care workers, the impact of an intervention program | 1. No control group  2. No clear description of intervention |
| Bennett  2003 | Learning circles: collaborating to promote RN and LPN role enhancement | 1. No control group |
| Lökk  2002 | Work site change and psychosocial well-being among health care personnel in geriatric wards | 1. Low quality assessment score |
| Petterson  2006 | Evaluation of an intervention programme based on empowerment for eldercare nursing staff | 1. No control group |
| Capuano  2004 | Work flow analysis. Eliminating non-value-added work | 1. No control group |
| Armstrong  2004 | Mandated staffing ratios, effect on nurse work satisfaction, anticipated turnover, and nurse retention in in an acute care hospital | 1. No control group |
| McGillisHall  2004 | Development and testing of quality work environments for nursing | 1. Low quality assessment score |
| Townsend  1991 | Creating a Better Work Environment | 1. No control group |
| Boumans  2004 | Differentiated practice, patient-oriented care and quality of work in a hospital in the Netherlands | 1. No pre test measure |
| Song  1997 | Nurses’ Job Satisfaction, absenteeism, and turnover after implementing a special care unit practice model | 1. No NWE (taxonomy) outcome measures |
| Bournes  2007 | Human becoming and 80/20: an innovative professional development model for nurses | 1. No control group  2. No NWE (taxonomy) outcome measures |
| Blenkarn  1988 | Primary nursing and job satisfaction | 1. No NWE (taxonomy) outcome measures |
| Forsgarde  2000 | Ethical discussion groups as an intervention to improve the climate in interprofessional work with the elderly and disabled | 1. No NWE (taxonomy) outcome measures |
| Boumans  1999 | Nurses’ well-being a in a primary nursing care setting in the Netherlands | 1. No NWE (taxonomy) outcome measures |
| Bernstein Hyman  1993 | Evaluation of an intervention for staff in a long-term care facility using a retrospective pretest design | 1. No control group  2. No pre and post measure design |
| Bourbonnais  2006 | Development and implementation of a participative intervention to improve the psychosocial work environment and mental health in an acute care hospital | 1. No control group |
| Harwood  2007 | Nurses’ perceptions of the impact of a renal nursing professional practice model on nursing outcomes, characteristics of practice environments and empowerment | 1. No control group |
| Zelauskas  1992 | The effects of implementing a professional practice model | 1. Low quality assessment score |
| Teasley  2007 | Improving work environment perceptions for nurses employed in a rural setting | 1. No control group |
| Gorman  1986 | Power and effective nursing practice | 1. Full article not available at the time |
