# Supplementary material for: Interventions aimed at improving the nursing work environment: a systematic review
Source: Implement Sci. 2010 Apr 27;5:34. doi: 10.1186/1748-5908-5-34 (PMC2876995; doi:10.1186/1748-5908-5-34)
Supplement: Additional file 3 — Characteristics of included studies. Detailed characteristics of included studies. [file 1748-5908-5-34-S3.DOC]

Additional file 3

Data extraction included studies

| **Sources** | **Design type** | **Intervention**  **Setting/format** | **Intervention Duration** | **Intervention Provider** | **Recipients of Intervention** | **Fidelity of Intervention** | **Content/**  **Elements** | **Outcome measures used** | **Quality score** |
| --- | --- | --- | --- | --- | --- | --- | --- | --- | --- |
| Berkhout *et al.* [23]  2004  The Netherlands | Pretest/post-test non-equivalent control group design with repeated measures | Setting: Dutch nursing homes Format:  individual | 22 months | The nursing homes were responsible for elaboration  and implementation of the model | **Age participants:** mean 31.7 **Gender:** 93% women **Educational levels:** Registered or enrolled nurse **N** = 147 | Not reported | Implementation of the resident oriented care model with assignment of patients to primary nurses | Resident assignment  Communication  Job autonomy  Job demands  Social support | Strong: 0,76 |
| Toloczko [26]  1989  United States | 3x2 factorial design, pretest/post-test control group design | Setting: hospital  Format: group | 6 weeks | 2 female licensed psychologists | **Age participants:** mean 37.6 **Gender:** 100% women **Educational levels:** registered nurses **N** = 47 | Not reported | Stress inoculation training (SIT) and social support training (SST) | Peer cohesion  Supervisor support | Moderate: 0,69 |
| Arnetz and Arnetz [32]  2000  Sweden | Experimental pre-post design with control group | Setting: emergency departments,  geriatric, psychiatric , and home healthcare sites  Format: individual and group | 1 year | Project coordinator, most often a supervisor. | **Educational levels:** Registered and practical nurses and practical nurses with special training in mental health **N** = 686 | Not reported | Structured program-regular discussion with staff to establish workplace routines for managing and reducing violent incidents directed towards healthcare staff | Registration of violent events directed towards healthcare workers | Moderate: 0,66 |
| Kennerly [25]  1996  United States | Quasi-experimental pre-post study with repeated measures | Setting: patients units of an acute care hospital  Format: individual | 18 months | Not reported | **Age participants:** mean 36 **Gender:** mean % of women 92.5 **Educational levels:** RN, LPN, nursing technicians, nursing aides, student nurse extends, secretaries (mean % of nurses 79) **N** = 115 | Not reported | Implementation of shared governance (an organizational framework for maximal participation in decisions about work and the workplace) | Co-worker support  Goal emphasis  Work facilitation  Interaction facilitation | Moderate: 0,65 |
| Arnetz and Hasson [29], 2007  Sweden | Non-randomized controlled study with repeated measures | Setting: municipal elderly care organizations  Format: individual | 3 years | A workgroup of researchers and elderly care management representatives | **Age participant:** ≤ 39 (mean 32.20%), 40-49 (mean 29.6%), ≥ 50 (mean 38.20%) **Gender:** 99% women **Educational levels:** Regular nurse (mean 64%), practical nurse (mean 55.6%), nurse’s aide (mean 38%) **N** = 270 | Not reported | A work group  collated a ‘‘toolbox’’ of practical instruments for  use at elderly care workplaces. Binders with information were distributed. | Leadership  Work climate  Participation  Goal clarity  Performance feedback  Skill’s development | Moderate: 0,65 |
| Mikkelsen *et al.* [27]  2000  Norway | Pretest/post-test control group design | Setting: community healthcare institution  Format: group | 1 year | External OD  facilitator/  consultant in concert with a ‘board’ (steering committee) | **Age participants:** Mean 42.5 **Gender:** Mean % of women 95.5 **Educational levels:** All of the supervisors and employees (nurses or assistant nurses) **N** = 76 | Limited assessment because employees were being confronted with restructuring and organizational changes | Participatory intervention for creating a learning process on how to identify and solve work problems in order to improve workplace health and organizational performance | Demands  Skill discretion  Decision authority | Moderate: 0,59 |
| Goodman [28] 1990  United States | Quasi-experimental study, non-equivalent control group design with pretest and posttest | Setting; nursing units in Medical centre hospital  Format: group | 14 weeks | The researcher was the NPQC facilitator | **Age participants:** Mean age NPC 33, mean age WU 36, mean age OU 35 **Gender:** All female except for one male **Educational levels:** Mean 66,7% RNs, mean 33.3% LPNs**N** = 63 | Not reported | Nursing practice quality circle: 2 educational sessions and 12 weekly meetings | Peer cohesion  Autonomy  Work pressure  Clarity  Control  Innovation  Physical comfort | Moderate: 0,59 |
| Eastburg *et al.* [31] 1994  United States | Pre-post design with control group | Setting: private medical hospital  Format: individual | One month | Researcher | **Age participants:** 39.3 **Gender:** 93 % women **Educational levels:** 3% master’s degrees, 30% bachelor’s degrees, 47% associate degrees, 20% high-school diploma **N** = 76 | Not reported | Positive-feedback  training for supervisors | Peer cohesion  Supervisor support | Moderate: 0,59 |
| Melchior *et al.* [24]  1999  The Netherlands | Quasi-experimental pre-post research design | Setting: long stay psychiatric care settings  Format: individual | 2,5 year | Nurse managers and quality care coordinators | **Age participants:** 34.8 **Gender:** 72% women **Educational levels:** Unit leaders, psychiatric nurses, practical nurses and nurses’ aids **N** = 176 | High drop-out rate and imitation of intervention by control group, necessitated additional analyses | Primary nursing. Nurse managers gave the primary nurses adequate feedback and support | Complexity  Autonomy  Feedback/clarity  Social leadership style  Instrumental leadership style | Moderate: 0,53 |
| Hallberg *et al.* [30] 1994  Sweden | Pretest/post-test control group design | Setting: Psychogeriatric clinic  Format: group | 1 year | Contact person, registered nurse and research assistant | **Age participants:** Mean age EW 32.9 and CW 38.4 **Gender:** 79.5% women **Educational levels:** Registered nurse (8), licensed mental nurse (23), licensed practical nurse (2), nurse auxiliary (2), no vocational training (4) **N** = 39 | Not reported | Individually planned and documented nursing care in combination with regular systematic clinical supervision | Recognition  Cooperation  Professional growth  Autonomy | Moderate: 0,53 |
| Gates *et al.* [33]  2005  United States | Quasi-experimental study, control group design with repeated measures | Setting: nursing homes  Format: group | 6 months | A masters-prepared nurse | **Age participants:** 36.0 **Gender:** 94% woman **Educational levels:** Nursing assistants  **N** = 138 | Not reported | Violence prevention intervention based on social cognitive theory with group sessions | Violence-prevention skills | Moderate: 0,53 |
