# Supplementary material for: Interventions aimed at improving the nursing work environment: a systematic review
Source: Implement Sci. 2010 Apr 27;5:34. doi: 10.1186/1748-5908-5-34 (PMC2876995; doi:10.1186/1748-5908-5-34)
Supplement: Additional file 4 — Summary of quality assessments. individual and summary quality assessment scores for each included study. [file 1748-5908-5-34-S4.DOC]

Additional file 4

Summary of quality assessments

| **Criteria** | **First author** | | | | | | | | | | | **Total** | |
| --- | --- | --- | --- | --- | --- | --- | --- | --- | --- | --- | --- | --- | --- |
|  | Berkhout  ^[23]^ | Toloczko  ^[26]^ | Kennerly  ^[25]^ | Arnetz and Arnetz ^[32]^ | Arnetz  ^[29]^ | Mikkelsen  ^[27]^ | Goodman  ^[28]^ | Eastburg  ^[31]^ | Gates  ^[33]^ | Melchior  ^[24]^ | Hallberg  ^[30]^ | **YES** | **NO** |
| **Sampling** |  |  |  |  |  |  |  |  |  |  |  |  |  |
| Use of probability sampling | N | N | N | N | N | N | N | N | Y | Y | N | 2 | 9 |
| Appropriate/justified sample size | Y | N | N | Y | Y | N | N | N | N | N | N | 3 | 8 |
| Sample drawn from more than one site | Y | N | N | Y | Y | Y | N | N | Y | Y | N | 6 | 5 |
| Response rate > 60% | Y | N | N | Y | Y | N | N | Y | N | N | Y | 5 | 6 |
| **Control of confounders** |  |  |  |  |  |  |  |  |  |  |  |  |  |
| Matching | N | Y | N | Y | N | N | N | N | N | N | Y | 3 | 8 |
| Statistical adjustment | Y | N | Y | N | Y | Y | Y | Y | Y | Y | N | 8 | 3 |
| Non matched | N | N | N | N | N | N | N | N | N | N | N | 0 | 11 |
| **Design:** |  |  |  |  |  |  |  |  |  |  |  |  |  |
| One pre-test or baseline and several post-test measures | Y | N | Y | N | Y | N | N | N | Y | N | Y | 5 | 6 |
| Simple before-and-after study | N | Y | N | Y | N | Y | Y | Y | N | Y | N | 6 | 5 |

| **Criteria** | **First author** | | | | | | | | | | | **Total** | |
| --- | --- | --- | --- | --- | --- | --- | --- | --- | --- | --- | --- | --- | --- |
|  | Berkhout  ^[23]^ | Toloczko  ^[26]^ | Kennerly  ^[25]^ | Arnetz and Arnetz ^[32]^ | Arnetz  ^[29]^ | Mikkelsen  ^[27]^ | Goodman  ^[28]^ | Eastburg  ^[31]^ | Gates  ^[33]^ | Melchior  ^[24]^ | Hallberg  ^[30]^ | **YES** | **NO** |
| **Measurement** |  |  |  |  |  |  |  |  |  |  |  |  |  |
| Reliability indices | Y | Y | Y | Y | Y | Y | Y | Y | Y | Y | N | 10 | 1 |
| Validity assessments | Y | Y | Y | Y | N | N | Y | N | N | N | N | 5 | 6 |
| **Statistical analysis** |  |  |  |  |  |  |  |  |  |  |  |  |  |
| Test are appropriate for the mean outcome and ≥ 80% of the others | Y | Y | Y | Y | Y | Y | Y | Y | Y | Y | Y | 11 | 0 |
| P values and confidence intervals are reported properly | Y | Y | Y | Y | Y | Y | Y | Y | Y | Y | Y | 11 | 0 |
| Correlations analyzed when multiple effects studied | Y | Y | Y | N | N | Y | Y | N | N | N | N | 5 | 6 |
| Missing data is managed appropriately | N | N/A | N | N | N | Y | N | N | N | N | N | 1 | 9 |
| **Drop outs** |  |  |  |  |  |  |  |  |  |  |  |  |  |
| Attrition rate < 30% | N | Y | Y | N | Y | N | Y | Y | N | N | N | 5 | 6 |

N = No Y = Yes N/A = Not applicable
